# Evaluation of self-esteem in adolescents of secondary school level

**DOI:** 10.1192/bjo.2021.635

**Published:** 2021-06-18

**Authors:** Farhana Begum, Usama Zubair, Iqbal Afridi, Fatima Toufique, Jawed Dars, Chunni Lal

**Affiliations:** 1 Patel Hospital; 2 Conolly Hospital; 3 Jinnah Postgraduate Medical Centre; 4 Sindh Rangers Hospital

## Abstract

**Aims:**

To assess the frequency of low self-esteem among adolescents of secondary school level in private schools of Karachi

**Method:**

It was a cross-sectional descriptive study done in ten private schools of Karachi for a period of 6 months. The proposal of the study was accepted by ethical committee of Jinnah Postgraduate Medical Centre, Karachi (called Institutional Review Board or IRB).The subjects fulfilling inclusion criteria were enrolled after obtaining informed consent by their parents. A semi-structured Performa was used to assess students’ particulars and included Rosenberg Self-esteem Questionnaire as a part of Performa. The data were analysed using SPSS version 17.0. Frequencies & percentages were generated for the level of self-esteem.Stratified analysis was done with a p value <0.05 taken as significant.

**Result:**

Out of the 246 students, 39.8% were of 14yrs of age, 36.2% were 15 years of age, while only 24% of adolescents were 16 years of age. Majority (64.2%) of the students were males while females were 35.8%. 70.3% of the students had normal level of self-esteem, whereas 28.9% of students had low self-esteem and only 0.8% of students had high self-esteem. Relationship of all the variables was found to be non-significant except that of educational level (p-value 0.047).

**Conclusion:**

Self-esteem was found to be normal in most of the adolescents of secondary school level but still more than 1/4th of the study participants had low self-esteem which if assessed and addressed early may save the individuals from mental health issues as well as problems at work and home life.Having a better understanding of self-esteem, can help us to identify the adolescents who have low self-esteem and are predisposed to develop mental health difficulties in future.It can lead to not only early intervention and reducing the burden of disease but also help in developing programs to help improve self-esteem in adolescents,hence increasing their overall motivation and productivity.

